# Exploring Herbal Medicine Use during Palliative Cancer Care: The Integrative Physician as a Facilitator of Pharmacist–Patient–Oncologist Communication

**DOI:** 10.3390/ph13120455

**Published:** 2020-12-11

**Authors:** Noah Samuels, Eran Ben-Arye

**Affiliations:** 1 Center for Integrative Complementary Medicine, Shaare Zedek Medical Center, Jerusalem 7791031, Israel; 2 Rappaport Faculty of Medicine, Technion-Israel Institute of Technology, Haifa 3525433, Israel; eranben@netvision.net.il; 3 Integrative Oncology Program, The Oncology Service, Lin, Zebulun, and Carmel Medical Centers, Clalit Health Services, Haifa 3535152, Israel

**Keywords:** oncology, pharmacist, integrative physician, complementary and integrative medicine, herbal medicine, safety, herb-drug interaction, multidisciplinary approach

## Abstract

Oncology patients frequently use herbal and other forms of complementary medicine, often without the knowledge of oncologists, pharmacists, and other healthcare professionals responsible for their care. Oncology healthcare professionals may lack the knowledge needed to guide their patients on the safe and effective use of herbal medicinal products, a number of which have potentially harmful effects, which include direct toxicity and negative herb–drug interactions. The current review addresses the prevalence and expectations of oncology patients from herbal medicine, as well as evidence for the beneficial or harmful effects of this practice (potential and actual), especially when the herbal products are used in conjunction with anticancer agents. Models of integrative oncology care are described, in which open and effective communication among oncologists, pharmacists, and integrative physicians on the use of herbal medicine by their patients occurs. This collaboration provides patients with a nonjudgmental and multidisciplinary approach to integrative medicine, echoing their own health-belief models of care during conventional cancer treatments. The role of the integrative physician is to facilitate this process, working with oncologists and pharmacists in the fostering of patient-centered palliative care, while ensuring a safe and effective treatment environment. **Case scenario:** W. is a 56 year old female artist who was recently diagnosed with localized hormone receptor-positive breast cancer. Following lumpectomy and sentinel node dissection, she is scheduled to begin adjuvant chemotherapy with a regimen which will include adriamycin, cyclophosphamide, and paclitaxel (AC-T protocol). She is worried about developing peripheral neuropathy and its impact on her ability to paint, and she asks about a number of dietary supplements which she heard could prevent this from happening: omega-3, vitamin E, alpha-lipoic acid, and acetyl-l-carnithine. She is concerned, however, that the supplements may negatively interact with her chemotherapy regimen.

## 1. Introduction

Patients with cancer often seek out nonconventional treatments for the relief of symptoms, as well as for disease-related outcomes, which include increasing survival, preventing disease recurrence, and “strengthening” their immune system [1,2,3]. The use of herbal medicine is one of the most popular of these modalities, with patients often believing that, because they are “natural”, these products are both effective and safe [4]. This belief is supported by the fact that many of the agents used in chemotherapy regimens are or were originally derived from botanical sources, such as paclitaxel (from the Pacific Yew tree, *Taxus brevifola*), etoposide/VP-16 (from the wild mandrake, *Podophyllum peltatum*), vinca alkaloids (from the rosy periwinkle, *Catharanthus roseus*), and others [5]. However, the herbal medicinal products which are being acquired and self-administered by oncology patients often lack scientific evidence for efficacy, and they may be accompanied by direct toxic effects or negative herb–drug interactions.

Despite the widespread use of herbal medicine among oncology patients, some with potentially harmful effects, it has been shown that, in as many as 50% of cases, these patients are not telling their oncology health care professional (HCP), especially oncologists and pharmacists, that they are doing so [6]. There are a number of reasons for this lack of disclosure, including the assumption that herbal medicine use is not relevant to their oncology treatment or that their oncology HCPs lack any knowledge on this practice and, therefore, cannot provide any guidance on their use [7]. Patients may also anticipate a negative or dismissive response from their oncology HCPs, although, in many cases, they are simply not asked [8,9]. It has been shown that many oncology HCPs are unaware of the potentially harmful outcomes associated with the use of herbal medicine during active cancer care, making it difficult for them to help their patients make an informed decision on the benefits and risks of this practice [10].

**Figure 1:**
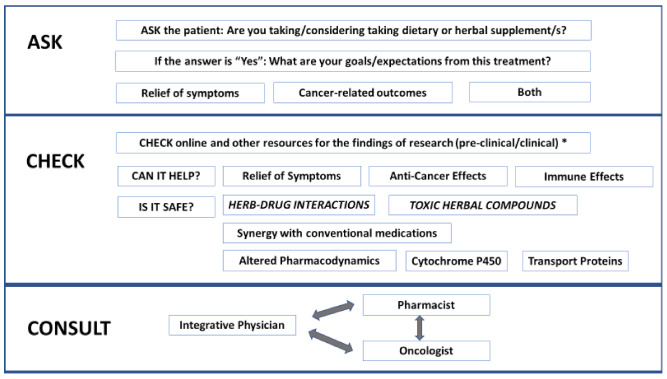
Algorithmic approach to addressing the use of herbal medicine by oncology patients (* differentiate between potential herb–drug interactions reported in preclinical research from actual interactions observed in the clinical setting).

The current review examines models of oncology care, in which oncologists and pharmacists can better address the use of herbal medicine by their patients. These models are invariably facilitated by integrative physicians who, working together with the patient’s oncology HCPs, provide guidance on the safe and effective use of herbal and other forms of complementary and integrative medicine within the supportive cancer care setting. The prevalence and expectations of patients from herbal medicine are addressed, as is the evidence for both beneficial and harmful effects of this practice (potential and actual), especially when used in conjunction with anticancer agents. The need for open and effective communication among oncologists, pharmacists, and integrative physicians is shown in these models to be both feasible and effective, facilitating a health-promoting and patient-centered therapeutic environment. An algorithmic approach to addressing the use of herbal medicine by oncology patients is outlined in Figure 1.

## 2. Anticancer Effects of Herbal Medicine

While research has shown that many herbal compounds consistently demonstrate significant anticancer activity, the overwhelming majority of this research has been conducted in the preclinical (in vitro, in vivo, and ex vivo) setting. Explanatory clinical research (randomized, controlled trials) of herbal medicine in the treatment of cancer is limited by a number of factors, which include difficulty in obtaining funding from private sources, since herbal compounds and formulas cannot be easily patented in most Western countries; a shortage of clinical researchers with an interest in an ability to conduct rigorous clinical trials in this particular field; and a reluctance on the part of conventional oncology departments to allow such research to take place in their centers, especially on patients participating in pharmaceutical company-funded research in which the use of herbal medicine is considered as an exclusion criterion.

In many cases, the significant anticancer activity of many herbal compounds observed in basic science studies is not translated into a similar effect in clinical research. An example of this is the active component of the spice turmeric (*Curcuma longa*, curcumin). A recent PubMed search using the keyword “curcumin” yielded nearly 16,000 references, with 6000 of them related to the treatment of cancer. However, only 70 of these papers are itemized as clinical research, and although the overwhelming majority of the preclinical studies show the significant anticancer activity of curcumin, clinical research has yet to show any statistically significant benefit of this herb on treatment outcomes or symptom load [11]. This discrepancy has been attributed to curcumin’s poor gastrointestinal absorption and rapid metabolism, which has prevented researchers from achieving what they believe to be therapeutic levels of the herbal compound in the patient’s serum [12]. The poor bioavailability of curcumin has been addressed with the use of nanotechnology particles which enhance its absorption [13], although there remains little if any clinical evidence supporting the use of this herb as an anticancer agent.

Another extensively researched herbal compound is mistletoe (*Viscum album*), a popular treatment among European oncology patients and a mainstay of anthroposophical medicine. Preclinical research has shown mistletoe to have significant ant-cancer activity, in both in vitro and in vivo settings [14]. In contrast to curcumin, clinical research has supported these findings, with a potential for significantly improving treatment outcomes in patients with advanced cancer. In a randomized controlled trial of 220 patients with locally advanced or metastatic pancreatic cancer, subcutaneous mistletoe injections were found to significantly increase survival rates when compared to no treatment [15]. However, further research is required before mistletoe injections can be incorporated into the conventional oncology treatment regimen for this purpose.

## 3. Herbal Medicine for Symptom Relief

A number of herbal medicinal products have been shown in rigorous clinical trials to be effective in helping relieve cancer treatment-related symptoms. These include ginger (*Zingiber officinale*) for chemotherapy-induced nausea and vomiting [16], ginseng (*Panax ginseng*, *P. quinquefolius*) for cancer-related fatigue [17], and mistletoe (*Viscum album*) for improved quality of life [15,18]. In a clinical study of patients with advanced non-small-cell lung cancer undergoing treatment with vinorelbine and cisplatin, the use of injections with the Chinese herb *Astragalus* (*Astragalus membranaceus*) was found to significantly improve quality of life and physical function; relieve fatigue, nausea and vomiting, and pain; and improve appetite. While a trend was observed for better treatment outcomes in the *Astragalus*-treated group, this finding was not shown to be statistically significant [19]. Moreover, a number of systematic reviews have concluded that herbal medicine can provide relief for symptoms related to classic chemotherapy regimens, such as the CMF protocol (cyclophosphamide, methotrexate and fluorouracil) in patients with breast cancer [20] and the FOLFOX regimen (fluorouracil, folinic acid and oxaliplatin) in patients with gastric [21] and colorectal cancer [22]

## 4. Safety of Herbal Medicine Use in Oncology

### 4.1. Legislation and Quality Control

Herbal and other dietary supplements are most often purchased by patients in community pharmacies or health food stores, without first consulting with a pharmacist or other medical professional [23,24]. In most Western countries, including the United States (US), the marketing and sale of dietary supplements do not require the manufacturer to show clinical efficacy, although legislation does require that the product be free from any toxic substances or conventional medication. Furthermore, while the law prohibits making any claim on the label regarding the ability of the herbal product to produce a beneficial clinical effect [25], many manufacturers are able to bypass this restriction by avoiding any explicit claims for a therapeutic effect or by providing a disclaimer on the label that the product is not being marketed as such. The sale of herbal products in pharmacies where conventional medications are sold may be seen by patients as an endorsement, despite the possibility (or probability) that these products do not actually help and may even cause harm.

The legislation in most countries also does not address the use of unproven and/or potentially harmful products with respect to the quantity and quality of the components listed on the product’s label. In a survey examining 50 herbal medicinal products with the word “ginseng” appearing on their labels, each product was tested for the presence and levels of the active component ginsenoside ((20*S*)-protopanaxadiol and (20*S*)-protopanaxatriol). It was discovered that six of the 50 products examined (12.0%) did not contain any active component whatsoever, while the remainder contained between 1.9% and 9.0% of the active component [26]. Standardization of herbal components is a complicated process, even more so when a number of herbal compounds are combined and sold as a formula, a common practice in traditional Chinese and other schools of medicine. Methods used to ensure batch-to-batch consistency of the herbal compound/s being investigated are mass spectrometry and high-performance liquid chromatography (HPLC). Spectrometry has been used to evaluate individual herbs [27,28], and HPLC has been used for herbal formulas containing a number of compounds, thereby providing reliable and reproducible findings regarding their safety and efficacy in the oncology setting (e.g., the herbal formulas PHY906 and LCS101 [29,30]).

### 4.2. Direct Toxic Effects of Medicinal Herbal Products

As with any conventional medication, herbal medicinal products have the potential to be accompanied by toxic and often significant adverse effects. Some of these may simply be difficult to digest, leading to gastrointestinal symptoms such as nausea and abdominal pain, commonly seen in patients ingesting wheat grass juice (*Triticum aestivum*) [31], omega-3 [32], and other supplements. Other herbal products may contain directly toxic components, although it is important to differentiate between potential toxicities, which are based on the findings of pre-clinical research, and true toxicities, as observed in clinical human research. An example of a severely toxic herbal product is laetrile, or “vitamin B17”. Laetrile is prepared from amygdalin, a compound extracted from the inner contents of fruit pits, primarily apricots. While claimed to be a “wonder cure”, a 1982 study showed that the product contained high levels of cyanide, with patients exhibiting overt signs of cyanide toxicity at high doses of the treatment (nausea and vomiting, headache and dizziness, mental obtundation, and dermatitis). At the same time, laetrile injections were not found to be effective in improving survival-related outcomes [33]. Following the publication of this study, laetrile and its related compounds were banned in most Western countries, although the product is still being sold as a cure for cancer on the internet.

### 4.3. Herb–Drug Interactions

The potential for a herbal compound to negatively interact with conventional cancer treatments is considered a major concern among many oncologists, pharmacists, and other oncology healthcare professionals. It is also an important question for patients and, as the case vignette indicates, may impact their decision as to whether they should start or continue taking the herbal product at all. Many of these patients are interested in receiving guidance on the effective and safe use of herbal medicine, especially those undergoing active anticancer treatment. In a study of 76 oncology patients undergoing chemotherapy, 78% were reportedly using a herbal vitamin supplement in conjunction with their conventional treatment regimen, with 27% identified as using supplements with potential interactions with a negative impact on the conventional treatment. The overwhelming majority of patients using herbal products (>85%) stated that they would discontinue their use or at least consult with their oncologist if they were to become aware of a potential for a harmful interaction with their conventional chemotherapy regimen [34].

Herb–drug interactions may occur through a number of pathways and, in many cases, more than one at the same time. These include medicinal effects of the herb and its compounds, which may be synergistic or antagonistic with the conventional medications the patient is taking, including anticancer agents, and/or alterations in drug pharmacodynamics, affecting bioavailability as a result of increased/decreased drug metabolism and absorption. Here, it is also important to differentiate between potential interactions, which are based on the findings of preclinical research, and true interactions, as observed in clinical human research.

#### 4.3.1. Synergistic/Antagonistic Effects

Herb–drug interactions can reflect a number of mechanisms, including synergistic or antagonistic effects with conventional drugs being used by the patient. For example, oncology patients are often treated with anticoagulant medications as a result of venous thrombosis, thought to be caused by the release of procoagulant factors by the tumor (Trousseau’s syndrome). Many herbal products contain compounds which have anticoagulant and/or antiplatelet properties, significantly increasing the risk for spontaneous bleeding in these patients [35]. Another example is medicinal mushrooms, which are popular among oncology patients and whose polysaccharides have been shown to reduce glucose levels and complications of diabetes mellitus [36]. However, medicinal mushrooms such as reishi (*Ganoderma lucidum*) and maitake (*Grifola fondosa)* have been associated with hypoglycemia in patients treated with antidiabetic drugs [37,38].

Herbal products may also inhibit the anticancer activity of chemotherapy agents. An example of this is the herb *Ephedra foemina* (“Alanda”), a herbal treatment for cancer which has become popular among the Arab-speaking population of northern Israel. The Alanda remedy is prepared by boiling water in a pot filled with vines from the locally ubiquitous ephedra plant and then drinking the brewed preparation. In an in vitro study, an XTT assay was used to test the cytotoxic activity of Alanda on two breast cancer cell lines (MDA-MB231 and SKBR3). While no anticancer activity of the herbal preparation (as measured by IC_50_) was detected, it was shown that the addition of Alanda to cancer cells in the presence of the chemotherapy agents carboplatin and cisplatin resulted in a dose-dependent reduction in their cytotoxic ability, by factors of 2 and 4 in the MDA-MB231 and SKBR3 breast cancer cell lines, respectively [27].Other herbs such as such as Japanese cornel (*Cornus officinalis*) and Indian sarsaparilla (*Hemidesmus indicus*) have been shown to have significant cytostatic effects on cancer cell lines [39,40], though their impact on conventional cytostatic agents remains unclear.

#### 4.3.2. Altered Pharmacodynamics

As with conventional drugs, herbal compounds have the potential to alter the pharmacodynamics of conventional anticancer drugs. The most well-known and researched of these is hypericum or St. John’s wort (*Hypericum perforatum*). Hypericum’s widespread use is a result of the extensive research showing its efficacy in the treatment of anxiety and depressive disorders [41]. However, as the use of this herb became more popular, physicians treating patients with kidney and heart transplants began to see more and more cases of organ rejection, despite the insistence of patients that they were diligent in their adherence to the antirejection immunosuppressant drug cyclosporin. It was discovered that patients showing signs of organ rejection had recently begun taking hypericum for depression and anxiety, which led to the discovery that the active component of the herb, hyperforin, was causing a significant reduction in cyclosporin serum levels. The mechanism for this effect involved two aspects of drug metabolism: induction of the cytochrome P450 enzyme CYP3A4, thereby increasing the metabolism of many drugs, including cyclosporin; and increased activity of p-glycoprotein (P-gp), a transport protein which inhibits gastrointestinal absorption of conventional medications. It was qshown that the ingestion of hypericum supplements, through the induction of the two enzymes, led to subtherapeutic plasma cyclosporine levels, with rejection of transplanted organs as a result [42,43].

The effects of hypericum on drug pharmacodynamics may have implications in the oncology setting as well. An example of this is the chemotherapy drug irinotecan (CPT-11), a commonly used agent in the treatment of gastrointestinal malignancies, usually in combination with fluorouracil (5-FU) and folinic acid (leucovorin; FOLFIRI protocol), often with the addition of oxaliplatin (FOLFIRINOX protocol). The concurrent use of hypericum with irinotecan was shown to lead to a reduction of 42% in serum levels of the drug [44]. This effect was found to be mediated by the pregnane X receptor (PXR), one of the main transcriptional regulators of CYP3A4 and P-gp [45]. A similar effect was seen when hypericum was used in conjunction with the protein tyrosine kinase inhibitor imatinib, with a resultant 43% increase in imatinib clearance, up to 32% lower mean area under the concentration–time curve (AUC), and significantly lower C-max and half-life (t_1/2_) of the drug [46]. Other examples include the compound naringnin, found in the juices of grapefruit and other citrus fruits, as well as apples. Naringin is a potent inhibitor of intestinal organic anion-transporting polypeptides (OATPs 1 and 2), decreasing exposure of co-administered substrates by as much as 85%) [47].

It is important to note that while the increase in drug metabolism induced by herbal compounds such as hypericum may lead to reduced serum levels, and thus the efficacy of anticancer agents, the reduction in drug metabolism may also have a negative effect on cancer treatment. An example of this is the drug tamoxifen (used by the patient in the case vignette), an estrogen antagonist which is the mainstay of treatment for hormone receptor-positive breast cancer. Tamoxifen is metabolized primarily in the liver by the cytochrome P450 enzymes CYP2D6 and CYP3A4 to the active metabolite endoxifen. Endoxifen is then excreted, primarily in the feces, following phase II metabolism through UDP-glucuronyltransferases (UGTs) and sulfotransferase (SULT) [48]. Herbs which reduce drug metabolism or inhibit second-pass metabolism could theoretically lead to reduced levels of endoxifen. The use of a curcumin supplement was found to reduce endoxifen levels in patients with breast cancer, through inhibition of the second-pass metabolism enzyme UGT [49].

#### 4.3.3. Potential vs. Real Herb–Drug Interactions

There is often a discrepancy between the potential for an herb–drug interaction, as shown in preclinical (in vitro, in vivo, and ex vivo) research, and what actually occurs in clinical practice. This discrepancy can be explained by the presence of a number of processes induced by the herbal compound affecting the bioavailability of the active components of the conventional drug. Curcumin is one of the most investigated herbal products being used by oncology patients, as mentioned earlier. Preclinical research has shown a wide range of effects induced by this herb on drug metabolism, many of which are contradictory to one another. For example, curcumin has been shown to increase CYP3A4 activity [50], reduce this enzyme’s activity [51], and both inhibit (intestinal) and induce (hepatic and renal) CYP3A4 activity, all without altering serum drug levels in a rat model [44,45]. Curcumin has also been shown to both increase and decrease P-gp activity, as well as that of the transport protein MDR-1 messenger RNA (mRNA) levels [52,53].

More often, it is unclear as to why certain herbs with clear effects on drug metabolism in preclinical research do not result in altered serum levels of the drug in the clinical setting. An example of this is the popular herb *Astragalus*, which has been shown to significantly induce pregnane X receptor (PXR)-regulated CYP3A4 transcription in HepG2 (human liver carcinoma) cells [54]. Yet, clinical research does not show any effect of *Astragalus* on serum levels of anticancer agents [55], including the drug docetaxel, an anticancer drug metabolized by the enzymes CYP3A4 and CYP3A5 in patients with non-small-cell lung cancer [56]. Another example is the green tea component epigallocatechin-3-gallate (EGCG), which significantly inhibits CYP3A4 activity and, thus, should reduce the metabolism of tamoxifen to its active component endoxifen, as mentioned in the previous section. Yet, in a randomized, controlled crossover trial of patients with breast cancer treated with tamoxifen, no difference was found in geometric mean endoxifen AUC 0–24 h in the period with green tea versus tamoxifen monotherapy (−0.4%; 95% CI, −8.6% to 8.5%; *p* = 0.92). Furthermore, no differences in C_max_ (−2.8%; 95% CI, −10.6% to 5.6%; *p* = 0.47) nor C_trough_ (1.2%; 95% CI, −7.3% to 10.5%; *p* = 0.77) were found [57].

## 5. A Multidisciplinary Approach to Guiding Oncology Patients on Herbal Medicine Use

There is an increasing number of oncology centers which are providing their patients with guidance on the safe and effective use of herbal medicine. These centers provide complementary medicine modalities to their patients, primarily with the goal of reducing symptoms and improving quality of life and function. Many of these integrative programs employ a multidisciplinary approach with the participation of oncologists, pharmacists, and integrative physicians.

### 5.1. The Role of the Oncologist

During the ongoing treatment of their patients’ malignancy, oncologists are expected to address concerns which are related to their symptom load and quality-of-life-related concerns. For many patients, this includes the provision of evidence-based guidance on the use of herbal medicine, including during their conventional anticancer treatment regimen. In this vein, the Clinical Oncology Society of Australia issued a position statement encouraging HCPs to initiate an open discussion with their patients on this practice [58]. In a cross-cultural survey of 770 Arab and Jewish oncology patients from northern Israel, both groups of patients stated that the primary expectation from their oncologist was to participate in formulating a complementary traditional medicine treatment plan, to be provided within the conventional oncology department [59].

While many oncologists agree that providing guidance to their patients on complementary medicine is an important service, most recognize that their knowledge in this field is limited. In an American Society for Clinical Oncology survey, it was shown that that fewer than half of the responding oncologists reported having initiated a discussion with their patients on the use of herbal and other dietary supplements. In addition, many of the oncologists questioned identified a lack of knowledge on nonconventional medicine as a major barrier to such a discussion [60]. In order to address the barriers preventing the integration of complementary medicine in the conventional oncology setting, a number of educational initiatives have been undertaken. In Israel, a course was offered to oncology physicians and nurses, in which they were taught about the philosophy and practice of complementary medicine modalities. The course also provided tools which the participating oncology HCPs could employ in order to establish an open and trusting dialogue with their patients about the use of these modalities [61]. In a project funded by the German Cancer Aid currently underway, the information needs of oncology patients and their healthcare professionals to help them make informed decisions on the use of complementary medicine are being addressed. The project includes the creation of a consulting manual for physicians, among other activities, and it is evaluating training programs in this field for oncology physicians, pediatric oncologists, and general practitioners [62].

### 5.2. The Role of the Pharmacist

As medication experts, pharmacists are in an ideal position to provide information about both conventional treatments and biological-based complementary medicine therapies [63]. There is a growing demand for oncology HCPs, including pharmacists, to be able to provide advice that ensures that these products are being used appropriately and safely [58,64]. However, it has been suggested that a lack of knowledge and education about the efficacy and safety of biological-based products act as a barrier to open discussions between HCPs and their patients [65]. In a survey of 70 Australian pharmacists examining perceptions, opinions, and knowledge about the use of complementary medicine by people living with cancer, it was estimated that 19% of daily inquiries were related to the use of biological-based complementary medicine. Furthermore, 72% of respondents believed they had a responsibility to advise about the concomitant use of these products with standard cancer treatments, despite 60% reporting a lack of confidence in their knowledge. The main barriers which were identified as preventing pharmacists from providing information about the use of these products included inadequate training on this subject (94%) and reservations about the evidence base for their efficacy and safety (50%) [63].

A qualitative study of pharmacists in China identified a number of barriers to the integration of herbal and other forms of traditional Chinese medicine by their patients in Western medicine practice. These included problems in the government’s addressing of this issue, as well as a lack of education and coordinated strategies among the government, education, pharmacy, pharmacist, and research sectors. Other barriers which were identified included a lack of clarity in defining the pharmacists’ role in this area and a disconnect between current regulatory standards and education/training systems [66]. In addition to their role in providing information to patients, it has been suggested that pharmacists need to identify psychosocial issues related to the patient’s use of herbal and other forms of complementary medicine, such as increased levels of anxiety [67].

### 5.3. The Role of the Integrative Physician

Integrative physicians are medical doctors who have undergone training in one or more complementary medicine modalities, incorporating these practices into their biomedical work [68]. The integrative physician can therefore understand the “language” of both conventional and complementary medicine, mediating between the often conflicting healthcare paradigms of the two and, thus, offering patients “the best of both worlds”. This is especially true for the oncology setting, where the integrative physician serves as a “gatekeeper”, providing patients and oncology HCPs with evidence-based guidance on the use of modalities such as herbal medicine, in both an effective and a safe environment.

In a survey of 269 female patients with breast cancer in Israel, 111 (41.3%) reported using herbal medicinal products for cancer-related goals. Among the entire cohort, 29.4% expected the integrative physician to provide guidance on the use of herbal medicine for cancer-related outcomes, with 48.6% of those using herbal medicine expressing this expectation (vs. 14.8% of nonusers; *p* < 0.001). The integrative physician recommended that 17 patients (15.3%) stop taking specific herbal products, because of potentially toxic effects and/or negative interactions with conventional drugs. At the same time, 10 patients in the entire cohort (3.7%) were advised to add a dietary/herbal supplement to their regimen, with the goal of reducing chemotherapy-related toxicities [69].

## 6. Integrative Models for Guiding Oncology Patients on the Use of Herbal Medicine

A number of models of oncology care which address the use of herbal medicine by patients have been described. Many cancer centers provide complementary medicine treatments as part of their supportive and palliative care service [70]. In the US, a number of leading oncology centers have services which provide their patients with a safe environment in which treatments such as herbal medicine are nontoxic and do not negatively interact with conventional anticancer treatment [71,72]. In an attempt to provide advisory services on herbal medicine use, a number of pharmacies have begun to employ naturopaths, with a recent survey among pharmacists with this service reporting that 64% of respondents valued the service and professionalism of the naturopaths in the community pharmacy [73]. In the US, the Naval Medical Center in San Diego conducts a monthly lecture in which oncology pharmacists review a patient’s prescription and herbal medicine profiles, identifying potential herb-drug interactions and, thus, preventing possibly harmful outcomes from the combination. This approach has been shown to enable both patients and healthcare professionals to make an informed decision about their use of herbal and other supplements [74].

In Israel, there are at present 11 active oncology and/or hemato-oncology complementary medicine programs: at the Lin, Carmel, Bnai Zion, Rambam, and Afulah medical centers in the north of the country; the Meir, Rabin, Tel Aviv/Ichilov, and Sheba medical centers in the central coastal region; and Shaare Zedek and Hadassah University Hospital medical centers in Jerusalem [75]. All of the Israeli integrative oncology services are headed by an integrative physician, with treatments performed by both them and nonmedical complementary medicine practitioners. In the majority of these centers (six of 10), guidance on the use of herbal medicine is provided either by an integrative physician or by a naturopath working with a multidisciplinary team of practitioners. At the Bnai Zion Medical Center, an ongoing discourse on the use of herbal medicine by patients takes place directly with the pharmacist, while at the Lin Medical Center the integrative physician consultation addresses the use of herbal medicine within a multimodal context which includes dietary changes, with patients offered active participation in an integrative oncology cuisine workshop [76].

## 7. Pharmacist–Oncologist–Integrative Physician Communication

In order to provide patients and their oncology HCPs with patient-oriented guidance on the use of herbal medicinal products during and after active cancer care, there needs to be effective communication among oncologists, oncology pharmacists, and integrative physicians. The interaction among these three participants in the patient’s care, with the patient in the center, needs to be studied far more than it has until now, both quantitatively and qualitatively. However, our experience has shown that this interaction can significantly promote patient wellbeing and increase the trust of patients and their caregivers in the oncology HCP team. The collaborative efforts of oncologists, pharmacists and integrative physicians can be even more effective when a number of practical steps are taken, using a list of questions to consider asking oncology patient when addressing their use of herbal medicine (Box 1). These steps can help identify herbal medicine use among oncology patients, encourage them and their caregivers to talk about their expectations from this practice, as well as their safety-related concerns, and facilitate a more open and informative communication with their team of oncology HCPs. Additional questions regarding the oncologist–pharmacist–integrative physician collaboration should also be taken into consideration, since they can enhance this collaboration and further promote the safe and effective use of herbal medicine by their patients (Box 2).

Box 1 **Questions to consider when discussing herbal medicine use with the oncology patient.**

* Are you presently taking/considering taking any dietary supplements or herbal medicinal products?

* Have you discussed the use of herbal medicine with your oncologist or oncology healthcare professional (pharmacist, nurse, other)?

* Is the goal of the treatment with herbal medicine to relieve your symptoms and improve quality of life? Is it to “fight” the cancer, prolong life, “strengthen” your immune system, or all of the above?

* Are you aware of any benefits from the use of herbal medicinal products (treating the cancer, reducing symptoms)? How about the potentially harmful effects of these products (toxic effects, negative herb–drug interactions)?

* Are you aware of the importance of both the quantity and the quality of the active components of herbal medicinal products? Have you thought about getting advice on this aspect of herbal medicine use?

* Have you considered consulting with an integrative physician who is familiar with nonconventional medicine, including the use of herbal medicinal products?

* After addressing the above questions, what are your expectations from the current interaction between us (patient–oncologist/pharmacist/integrative physician)? How would you like me to be of further assistance? Do you feel that I am able/unable to answer your questions about the use of herbal medicine?

Box 2 **Questions to consider about the oncologist–pharmacist–integrative physician collaboration regarding herbal medicine use among oncology patients.**

* Are you interested in undergoing training/further training in herbal medicine? If so, what would be the goals of this process? Would it include understanding the indications and evidence for the effectiveness of herbal medicine in the relief of oncology-related symptoms; contraindications of specific herbal medicinal products; quality/quantity control of the herbal medicinal products being used by patients; and/or resources for further training which will meet your expectations regarding what you need to know about the use of herbal medicine in oncology?

* Would you be interested in working with/joining an integrative oncology team/participating in the consultation on herbal medicine use? Who are the professionals which you feel would be most helpful for this goal (integrative physician, naturopath, dietician, herbalist)? How can you locate an integrative medicine practitioner/physician who can provide a high level of complementary medicine, including the use of herbal medicine, to your multidisciplinary team of oncology healthcare professionals?

* In which kind of setting would you like to implement your training in herbal medicine, within your multidisciplinary team of oncology healthcare professionals? Would it be in the public/private sector or a research/academic-focused setting?

* How much do you wish to become involved in the clinical treatment of the patient? How much time can you dedicate to working in an integrative capacity, including providing guidance on herbal medicine use by your oncology patients?

## 8. Conclusions

Healthcare professionals who treat oncology patients need to be aware that many of them are using herbal medicinal products, often without their knowledge. In order to effectively address the benefits and risks of this practice, oncologists, pharmacists, and integrative physicians need to work together and communicate effectively, conducting open and nonjudgmental conversations with their patients about this practice. In this way, informed and evidence-based decisions can be made, and the patient can be monitored for any untoward effects of the nonconventional treatment. While some herbal medicinal products can provide relief from symptoms, they may also result in harmful effects which include direct toxicity and herb–drug interactions.

The multidisciplinary approach can increase the likelihood that oncology patients are using herbal medicine in a safe and effective fashion. With oncologists, pharmacists, and integrative physicians working together for this purpose, patients will most likely be more willing to disclose their current or planned use of herbal medicinal products, facilitating a nonjudgmental and patient-centered conversation about this practice. For this to occur, integrative physicians should be included as part of the conventional oncology staff, serving as “gatekeepers” with respect to the use of nonconventional modalities, identifying the use of herbal and other complementary medicine modalities, and providing evidence-based guidance on the safe and effective use of these practices. Further research of the multidisciplinary collaboration is needed to better understand the implications of the sum of the parts, as opposed to each discipline (oncologist, pharmacist, and integrative physician) on its own.
